# Clinical Impact of COVID-19 on Multi-Drug-Resistant Gram-Negative Bacilli Bloodstream Infections in an Intensive Care Unit Setting: Two Pandemics Compared

**DOI:** 10.3390/antibiotics11070926

**Published:** 2022-07-09

**Authors:** Francesco Cogliati Dezza, Gabriele Arcari, Federica Alessi, Serena Valeri, Ambrogio Curtolo, Federica Sacco, Giancarlo Ceccarelli, Giammarco Raponi, Francesco Alessandri, Claudio Maria Mastroianni, Mario Venditti, Alessandra Oliva

**Affiliations:** 1 Department of Public Health and Infectious Diseases, Sapienza University of Rome, 00185 Rome, Italy; francesco.cogliatidezza@uniroma1.it (F.C.D.); federica.alessi@uniroma1.it (F.A.); serena.valeri@uniroma1.it (S.V.); ambrogio.curt@uniroma1.it (A.C.); giancarlo.ceccarelli@uniroma1.it (G.C.); caludio.mastroianni@uniroma1.it (C.M.M.); mario.venditti@uniroma1.it (M.V.); 2 Microbiology and Virology Laboratory, Department of Molecular Medicine, Sapienza University of Rome, 00185 Rome, Italy; gabriele.arcari@uniroma1.it (G.A.); federica.sacco@uniroma1.it (F.S.); giammarco.raponi@uniroma1.it (G.R.); 3 Department of Anesthesia and Critical Care Medicine, Sapienza University of Rome, Policlinico Umberto I, 00161 Rome, Italy; f.alessandri@policlinicoumberto1.it

**Keywords:** antibiotic therapy, multi-drug-resistant bacteria, COVID-19, bloodstream infections, intensive care unit

## Abstract

Two mutually related pandemics are ongoing worldwide: the COVID-19 and antimicrobial resistance pandemics. This study aims to evaluate the impact of COVID-19 on multi-drug-resistant Gram-negative bacteria (MDR-GN) bloodstream infections (BSIs) in a single intensive care unit (ICU). We conducted a retrospective study including patients admitted to the ICU, reorganized for COVID-19 patients’ healthcare, with at least one confirmed MDR-GN BSI during 2019–2020. We compared clinical and microbiological features, incidence density, antibiotic therapy and mortality rate in pre- and during-COVID-19 pandemic periods. We estimated the impact of COVID-19 on mortality by means of univariate Cox regression analyses. A total of 46 patients were included in the study (28 non-COVID-19/18 COVID-19). Overall, 63 BSI episodes occurred (44/19), and non-COVID-19 patients had a higher incidence of MDR-GN BSIs and were more likely to present *K. pneumoniae* BSIs, while the COVID-19 group showed more *A. baumannii* BSIs with higher per pathogen incidence. COVID-19 patients presented more critical conditions at the BSI onset, a shorter hospitalization time from BSI to death and higher 30-day mortality rate from BSI onset. COVID-19 and septic shock were associated with 30-day mortality from MDR-GN BSIs, while early active therapy was a protective factor. In conclusion, COVID-19 showed a negative impact on patients with MDR-GN BSIs admitted to the ICU.

## 1. Introduction

Since 2020, two mutually related pandemics are ongoing worldwide: COVID-19 and antimicrobial resistance (AMR) pandemics. The former is a big global threat that has resulted, at the time of writing, in over 6 million deaths [1] and disrupted healthcare systems at all levels, leading to a reorganization of health facilities and changes in social behavior all over the world. The second, much less visible and known, is also called a «hidden pandemic», and it has been recognized as one of the most urgent threats facing global health, economic and social development, causing over 1 million estimated deaths attributable to AMR bacteria in 2019 [2,3,4].

From the beginning of the SARS-CoV-2 pandemic, experts have warned about the relation between COVID-19 and AMR and suggested increasing Infection Prevention and Control (IPC) measures and antimicrobial stewardship programs to avoid unnecessary antibiotic use to reduce the potential long-term impact on AMR [5,6]. Nevertheless, the gap between the prevalence of antibiotic prescribing (~75%) and the estimated bacterial co-infection (8.6%) upon admission of COVID-19 patients requiring hospitalization was largely reported, thus leading to a high level of inappropriate antimicrobial exposure [7].

Studies reported conflicting evidence on the impact of COVID-19 on multi-drug-resistant organism (MDRO) infections. Several reports—particularly from Italy, Brazil, and Germany—described outbreaks or significant increases in MDRO infections during the COVID-19 period [8,9,10]. However, other studies from Spain and France did not describe an increase in MDR infections [11,12], and, interestingly, an Irish study showed a decrease in hospital-acquired *Clostridioides difficile* infections compared to the pre-COVID-19 pandemic period [13]. 

As for critically ill patients admitted to intensive care units (ICUs) during the COVID-19 pandemic, few papers have investigated the burden of MDRO, especially for Gram-negative bacteria invasive infections such as bloodstream infections (BSIs) [9,14,15,16]. In a review in 2020, Fattorini et al. found that only 1.3% of 522 COVID-19 patients in the ICUs developed an infection from MDR bacteria [17], while another review showed a prevalence of carbapenem-resistant *Klebsiella pneumoniae* that ranged from 0.35% to 53% [18]. As a matter of fact, many hospitals have reported MDRO outbreaks in the ICU during the COVID-19 pandemic, mostly caused by Gram-negative bacteria or *Candida auris* [19]. Carbapenem-resistant *Acinetobacter baumannii* (CRAB) seems to be the main pathogen involved with a higher incidence compared to the pre-COVID-19 period and a higher risk of death than other MDROs [20,21,22].

In order to add new findings regarding the burden of MDRO infection in SARS-CoV-2-infected, critically ill patients, this study aimed to evaluate the impact of COVID-19 on MDR Gram-negative bacteria BSIs (MDR-GN BSIs) in a single ICU reorganized for exclusive COVID-19 patients’ healthcare.

## 2. Results

During the study period, 63 (44 pre-pandemic (no-CoV) and 19 pandemic (CoV)) BSI episodes were observed in 46 patients (28 no-CoV and 18 CoV). The demographic and clinical characteristics of the study population with the microbiological data and clinical characteristics of the BSI episodes are summarized in Table 1. No differences between no-CoV and CoV patients were observed regarding age, gender, previous hospitalization, previous antibiotic therapy, and the burden of comorbidities, calculated through CCI.

Instead, the no-CoV population was more likely to present a higher APACHE II score at ICU admission than the CoV population (median 22 (IQR 16–23) vs. 11 (3.2–21.5), respectively, *p*: 0.003), to get admitted to the ICU for polytrauma (9 (32.1%) vs. 0 (0), *p*: 0.007), and to show more than one BSI episode from different MDR species (13 (28.3%) vs. 1 (2.2%), *p*: 0.003).

### 2.1. Microbiological Data

The overall MDR-GN BSI incidence density for the pre-COVID19 period was 16.5 per 100 patients and 11.2 per 100 patients for the COVID-19 period (*p*: 0.12). In the pre-COVID-19 period, the *K. pneumoniae* BSI incidence density was 8.2 per 100 patients, and the *A. baumannii* BSI incidence density was 6.4 per 100 patients. Instead, during the COVID-19 pandemic, the *K. pneumoniae* BSI incidence density was significantly lower (2.4 per 100 patients, *p*: 0.012), and the *A. baumannii* BSI incident density was higher (9.0 per 100 patients, *p*: 0.34).

As for the BSI episodes, no differences were observed regarding the BSI focus, source control, early active therapy, and the time to definite therapy. However, CoV patients presented significantly more critical conditions at the BSI onset with higher PITT scores (8 (2–8) vs. 3 (1–5), *p*: 0.002) and more frequently had septic shock (13 (68.4%) vs. 12 (27.3%), *p*: 0.003) than no-CoV patients. In contrast, the no-CoV group was more likely to show an infection before BSI onset (pre-BSI infection) from both no-MDRO (*p*: 0.003) and MDRO (*p*: 0.004), although no differences were observed in antibiotic therapy performed before the BSI onset.

MDRO colonization before the BSI onset (36 (81.8%) vs. 9 (47.4%)) and *K. pneumoniae* BSI (22 (50%) vs. 4 (21%)) were significantly more frequent in no-CoV patients (*p*: 0.005 and *p*: 0.032, respectively); on the other hand, the CoV group showed more *A. baumannii* BSIs (15 (78.6%) vs. 17 (38.6%), *p*:0.003). Difficult-to-treat resistant (DTR) *P. aeruginosa* was only observed in five no-CoV patients. As for the local epidemiology in the ICU, MDR *K. pneumoniae* almost exclusively presented KPC genes (twenty-four) except for one NDM+ and one OXA-48+ strain.

### 2.2. Hospitalization and Mortality Data

No-CoV patients showed a significantly longer overall hospital stay (*p*:0.001) as well as in the ICU (*p*: 0.004) and a longer time from hospital admission to BSI onset (*p*: 0.02), whereas CoV patients presented a shorter hospitalization time from BSI onset to death (*p*: 0.003). No differences were observed in the days from ICU hospitalization to BSI onset (Table 1).

**Figure 1:**
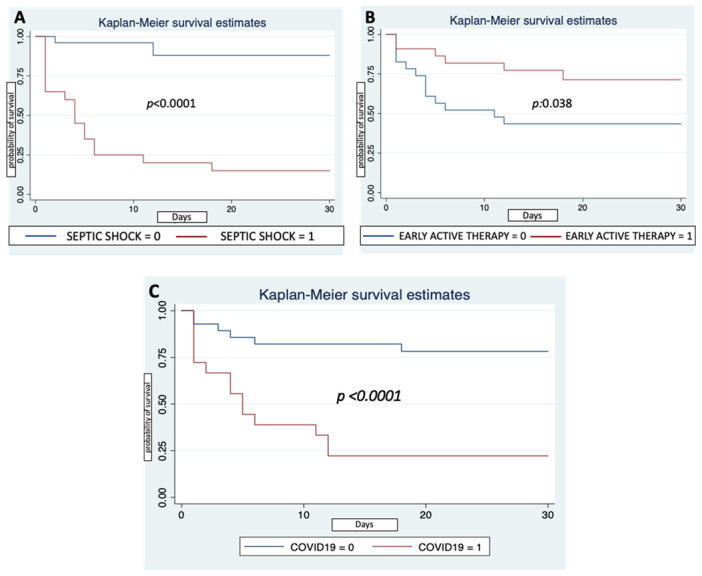
Cumulative proportions on overall 30-day mortality estimates from MDR-GN BSI onset. (**A**), between patients with (red line) or without (blue line) septic shock at the BSI onset; (**B**), between patients that received early active therapy (red line) and who did not receive it (blue line); (**C**), between pre-pandemic patients (blue line) and COVID-19 patients (red line). Axis X: number of days from BSI onset; axis Y: probability of survival from BSI onset. *Abbreviations*: MDR-GN: multi-drug-resistant Gram-negative bacteria; BSI: bloodstream infection; early active therapy: at least one in vitro active drug within the first 24 h.

Patients that presented more critical conditions at the BSI onset with documented septic shock had a higher 30-day mortality rate from BSI onset; *p* < 0.0001 (Figure 1A). Meanwhile, patients that underwent an early active therapy (as at least one in vitro active drug within the first 24 h) showed a higher survival rate; *p*: 0.038 (Figure 1B).

Finally, CoV patients had a higher 30-day mortality rate from ICU admission (14 (77.8%) vs. 6 (21.4%), *p* < 0.0001) and from BSI onset (14 (77.8%) vs. 6 (21.4%), *p* < 0.0001), as shown in Figure 1C.

## 3. Discussion

In the present study, we showed that COVID-19 had a negative impact on patients with MDR-GN BSIs admitted to the ICU. We also confirmed the prognostic role of severity at ICU admission and at the BSI onset and confirmed the importance of *A. baumannii* as a causative agent of BSIs during the COVID-19 pandemic.

In fact, during the COVID-19 pandemic, the impact on AMR has been more pronounced inside ICU settings [19,23]. These observations are supported by the evidence that antibiotics prescription was higher in ICUs than in general wards, peaking at more than 86% among critical patients and therefore leading to unnecessarily high antimicrobial use [7]. Furthermore, other contributing factors to AMR could be the patients’ critical illness, inappropriate adherence to IPC protocols, personal protective equipment shortages, critically low healthcare workers/patients ratios with overcrowded wards, and the disruption of MDRO screening and surveillance programs as well as antimicrobial stewardship activities [19,23]. 

However, how the incidence of MDRO infections changed during the pre-COVID-19 and during-COVID-19 periods is still debated. We found that the overall MDR-GN BSI incidence density was lower in the CoV patients than the no-CoV ones, although the rate per pathogen showed an increase in the CRAB incidence in the former group of patients, albeit not significant. Several studies reported a decrease or no change in the incidence of MDRO colonization or infection in ICUs during the COVID-19 pandemic period, as with our findings [9,21,24,25], while other studies observed a significant increase in MDRO infections [15,20,22] or an increase only in a specific subgroup of patients with colonization or infection due to CRAB, as our study showed [9,21]. The change in our local epidemiology is associated with some outbreaks of CRAB infection during the observation period which reduced the *K. pneumoniae* incidence density. Furthermore, the higher level of CRAB pneumonia in the CoV patients as a source of BSIs, although not significant, could explain this epidemiology alternation, but the real explanation is not fully elucidated. Of interest, the burden of *A. baumannii* infections in CoV patients has been described worldwide with several outbreaks reported [19]. This so-called “little pandemic” within the huge COVID-19 pandemic represents a matter of concern as the greatest threat to COVID-19 patients admitted to ICUs [9,20,21,22,26]. However, new observational studies are needed to assess the real impact of CRAB invasive infections on mortality in critically ill CoV patients [20,22,26].

Among critically ill COVID-19 patients, BSIs are one of the major secondary infections often caused by Gram-negative bacteria [27,28]; moreover, several studies showed an increase in MDR-GN BSI during the COVID19 pandemic. Nevertheless, the real burden of these invasive infections and how they impact on mortality have not been fully estimated yet [14,20,22,26].

Despite the remarkable difference in patients’ severity (the no-CoV group showed more critical conditions at ICU admission), mortality was higher in the CoV patients. This result is comparable to other previous reports from Italy and France, in which a higher in-hospital mortality rate was observed in patients with SARS-CoV-2 infection admitted to ICUs [14,20]. In particular, our experience showed that, although the two groups did not significantly differ in days from ICU admission to BSI onset, CoV patients presented a much shorter survival time after BSI onset than no-CoV patients, as shown in Figure 1C. 

This observation joined with the more severe clinical condition of CoV patients at the BSI onset (with higher PITT scores and more frequent septic shock) seems to suggest that MDR-GN BSIs might represent only the last fatal complication in patients with an already ominous prognosis. Nevertheless, we confirmed that early active therapy represents an important factor associated with a better outcome in critically ill patients with MDR-GN BSIs [29,30,31]. Therefore, additional studies are warranted in order to fully elucidate whether the BSI onset impacts the outcome of CoV patients or if it represents just the “last” event in patients with a poor prognosis per se.

CoV patients presented a significantly lower MDRO colonization but a more frequent primary BSI than the no-CoV group, although this did not reach statistical significance. These results could indirectly confirm gut damage, microbiota variation and persistent microbial translocation during SARS-CoV-2 infection, which is more pronounced in severe infections and independent from gastrointestinal manifestations [32,33]. In this context, the observed gut mucosal perturbation may play a pathogenetic role in the development of BSIs following infections [34,35].

Our study has several limitations. First of all, it was retrospective nature and had a small population. So, due to the small population and some residual confounder factors, we decided to not perform a powerful statistical assessment via multivariable analysis. Secondly, we did not recollect data about the immunosuppressive treatment that could affect the different outcomes of our patients. Thirdly, the monocentric study reflected the ecology and epidemiology of our hospital which could differ from other ICUs; therefore, our results might not be generalized. Finally, we only focused on bacteremia, and we did not recollect data from other MDR infections; accordingly, our report may underestimate the real AMR burden pre- and during the COVID-19 pandemic.

## 4. Materials and Methods

Over two years (2019–2020), we conducted an observational, retrospective, single-center study including patients admitted to the same ICU of an academic tertiary hospital in Rome, with at least one confirmed MDR-GN BSI.

**Figure 2:**
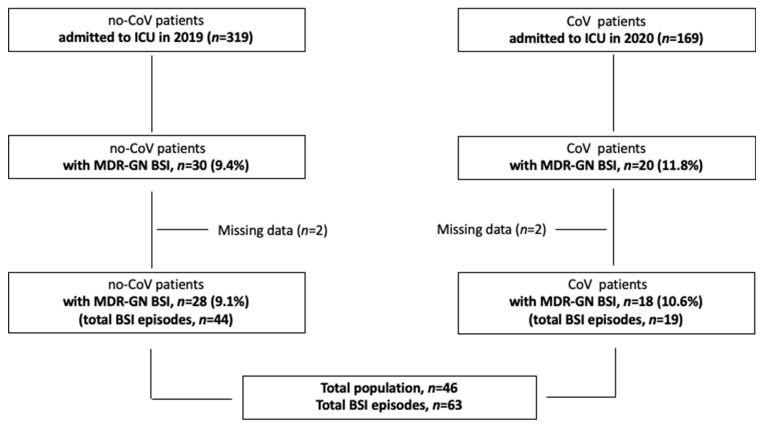
Flow chart of the study population. *Abbreviations*: no-CoV: pre-COVID-19 pandemic period; CoV: during COVID-19 pandemic; ICU: intensive care unit; MDR-GN: multi-drug-resistant Gram-negative bacteria; BSI: bloodstream infection.

All clinical data were systematically analyzed from patients admitted in the pre-COVID-19 period (March–December 2019) and during (March–December 2020) the first COVID-19 pandemic period in a single ICU which, in March 2020, was reorganized for exclusive COVID-19 patients’ healthcare. Patients were enrolled in the study if they fulfilled the following inclusion criteria: (I) age > 18 years, (II) confirmed MDR-GN BSI, and (III) confirmed SARS-CoV-2 infection via polymerase-chain-reaction assay on nasopharyngeal swab only during the COVID-19 period. Only the first BSI from the same MDR-GN species was included in the analyses. The flow chart of the study population is represented in Figure 2.

We reviewed patient data from medical records and anonymously recorded the following information in an electronic database: demographics, comorbidities, clinical and laboratory findings at the ICU admission and at the BSI onset, microbiological data during ICU hospitalization, antibiotic treatments and procedures administered during hospitalization and/or in the 90 days prior to BSI onset, source of BSI, duration of hospitalization and ICU stay, and time to BSI onset and 30-day mortality from ICU admission and from BSI onset.

### 4.1. Definitions

The patients admitted to the ICU in the pre-COVI-19 period and during the COVID-19 pandemic were named no-CoV and CoV, respectively.

Patients’ severity at ICU admission was defined by the APACHE II score that collected these characteristics: history of severe organ failure, age, body temperature, mean arterial pressure, pH, heart and respiratory rate, sodium, potassium, creatinine, acute renal failure, hematocrit, white blood cell count, Glasgow Coma Scale and FiO2 [36], whereas the burden of comorbidities was estimated by means of the Charlson Comorbidity Index (CCI) [37]. The severity of BSI was defined according to the PITT score that collected these characteristics: body temperature, arterial pressure and need for vasopressor, mechanical ventilation, cardiac arrest, and mental status [38]. Septic shock was defined according to the international consensus [39]. BSI onset was considered the date of the index blood culture collection.

According to the hospital’s guidelines, rectal/stool swab culture was routinely evaluated for MDRO strains, and an MDRO colonization was defined as a rectal/stool swab positive culture in the absence of clinical signs of infection. MDR bacteria was defined according to the classification of Magiorakos et al. [40], while DTR *P. aeruginosa* was defined as in the IDSA guidelines 2022 [41]. Primary bloodstream infection was defined as a BSI occurring in patients without a recognized source of infection.

The source control was achieved when all those physical measures used to control the focus of BSI and to restore the optimal function of the affected area were performed.

Early active therapy was defined as the use of at least one in vitro active drug within the first 24 h from the BSI onset, and the time to definite therapy was days from BSI onset to the definite therapy. All infections caused by MDR or non-MDR bacteria that occurred before the BSI were defined pre-BSI infection from MDRO or nno-MDRO, respectively.

### 4.2. Microbiological Analyses

Blood culture bottles were incubated in the automatic VirtuoBacT/Alert system (bioMérieux, Inc., Marcy l’Etoile, France). Isolated colonies from blood cultures were identified using the Matrix-Assisted Laser Desorption Ionization–Time Of Fight Mass Spectrometry (MALDI-TOF MS) system (Bruker Daltonik GmbH, Bremen, Germany). Antimicrobial susceptibility was tested using the MicroScan WalkAway system (Beckman Coulter, Inc., Brea, CA, USA). The MICs of antibiotics were assessed by following EUCAST breakpoint tables for the interpretation of MICs and zone diameters, version 9.0, valid from 2 January 2019 [42].

Strains showing a carbapenem-resistant phenotype (according to EUCAST criteria [42]) were tested using the real-time PCR assay Xpert Carba-R kit for the GeneXpert system (Cepheid, Sunnyvale, CA, USA) to evaluate the presence of the blaVIM, blaIMP, blaKPC, blaOXA-48, and blaNDM carbapenemase genes.

#### Statistical Analysis

The data were given as medians with interquartile ranges (IQR, 25th–75th percentile) for continuous variables and as simple frequencies, proportions, and percentages for categorical variables. The Mann–Whitney test was used for unpaired samples. Dichotomous variables were compared using Fisher’s exact tests or chi-square test statistics, as appropriate. Log-rank tests and univariate Cox regression were used for categorical or continuous variables, respectively. *p*-value analyses were two-sided, and a *p*-value of less than 0.05 was considered statistically significant.

The rates of MDR-GN BSI in the pre-COVID-19 period and during the COVID-19 pandemic were expressed in incidence densities which were calculated using the number of MDR-GN BSI episodes in the study period as the numerator and the number of patients admitted in the ICU in the same period as the denominator multiplied by 100. We analyzed total rates and rates per pathogen in the ICU. To assess the differences between the two periods, the incidence densities in 2019 were compared with the data from 2020. All statistical analyses were performed with SATA/IC software (StataCorp) version 15. 

The study was approved by the local Ethics Committee (ID Prot. 109/2020).

## 5. Conclusions

COVID-19 had a negative prognostic impact on patients with MDR-GN BSI admitted to the ICU. Enhancing IPC measures is crucial to reducing the likelihood of MDRO colonization and bacteremia, which is related to a worse outcome. Further studies are needed to clearly assess the relationship between COVID-19 and AMR.

## Figures and Tables

**Table 1:** **Characteristics of the study population.** Demographic, clinical characteristics, outcomes of the study population and microbiological data, and clinical and treatment characteristics of the BSI episodes.

Characteristics	Total Population	No-CoV	CoV	*p* Value
	(*n* 46)	(*n* 28)	(*n* 18)	
General, *n*, %				
Age, years, median (IQR)	65.5 (57–73)	66.5 (56.7–73)	63.5 (57.5–72.5)	ns
Gender, F/M	14 (30.4)/32 (69.6)	11 (39.3)/17 (60.7)	3 (16.7)/15 (83.3)	ns
Other hospitalization in previous 90 days	10 (21.7)	8 (22.8)	2 (10.5)	ns
Pre-hospitalization antibiotic therapy in previous 90 days	13 (28.3)	6 (20.4)	7 (36.8)	ns
Pre-BSI antibiotic therapy	45 (97.8)	27 (95.4)	18 (100)	ns
Patients with >1 BSI from different MDRO	14 (30.4)	13 (28.3)	1 (2.2)	0.003
**Comorbidities, *n*. (%)**				
Myocardial infarction	7 (15.2)	5 (17.9)	2 (11.1)	ns
Congestive heart failure	14 (30.4)	10 (35.7)	4 (22.2)	ns
Peripheral vascular disease	13 (28.3)	8 (28.6)	5 (27.8)	ns
Cerebrovascular disease	4 (8.7)	3 (10.7)	1 (5.5)	ns
Dementia	3 (6.5)	2 (7.1)	1 (5.5)	ns
Chronic obstructive pulmonary disease	6 (13)	6 (21.4)	0	NA
Liver disease {1}	1 (2.2)	1 (3.6)	0	NA
Diabetes mellitus	11 (23.9)	8 (28.6)	3 (7.2)	ns
Hemiplegia	1 (2.2)	1 (3.6)	0	NA
Chronic kidney disease {2}	2 (4.4)	1 (3.6)	1 (5.5)	ns
Solid tumor	3 (6.5)	3 (10.7)	0	NA
Leukemia	1 (2.2)	0	1 (5.5)	NA
Charlson Comorbidity Index, median (IQR)	3.5 (2–6)	4 (2–6)	2 (1.25–4)	ns
APACHE II score {3}, median (IQR)	18.5 (13–23)	22 (16–23)	11 (3.2–21.5)	0.003
Polytrauma	9 (19.6)	9 (32.1)	0 (0)	0.007
**Outcome, median (IQR)**				
Days of hospitalization overall	43 (29.5–98.75)	69.5 (43.5–141.5)	28.5(18.7–35.7)	0.001
Days of hospitalization until ICU admission	2 (0–6)	2 (0–9.75)	2 (0.25–5)	ns
Days of hospitalization until BSI	25.5 (12.25–37.5)	29 (15–48.25)	20 (9.5–26.75)	0.02
Days of ICU hospitalization	31.5 (21.25–68)	56 (28–91.25)	25 (13.5–29.5)	0.004
Days of ICU hospitalization until BSI	18.5 (9–29)	23 (9.75–34.75)	14.5 (9–21.75)	ns
Days of hospitalization from BSI to death	15.5 (4.25–45.75)	44 (14.5–117.5)	5 (1.25–11.75)	0.003
Overall in-hospital mortality, *n*. (%)	29 (63)	15 (53.6)	14 (77.8)	ns
Mortality 30 days from ICU admission, *n*. (%)	20 (43.5)	6 (21.4)	14 (77.8)	<0.0001
Mortality 30 days from BSI, *n*. (%)	20 (43.5)	6 (21.4)	14 (77.8)	<0.0001
**BSI Characteristics**	**Total BSI**	**No-CoV**	**CoV**	
	**(*n* 63)**	**(*n* 44)**	**(*n* 19)**	
**Microbiological data, *n* (%)**				
Pre-BSI infections from no MDRO {4}	57 (90.6)	43 (97.7%)	14 (73.7)	0.003
Pre-BSI infections from MDRO {4}	48 (76.2)	38 (86.4)	10 (52.6)	0.004
MDRO colonization	45 (71.4)	36 (81.8)	9 (47.4)	0.005
Overall BSI incidence density, *n* per 100 patients		16.5	11.2	ns
*K. pneumoniae* BSI	26 (41.3)	22 (50)	4 (21)	0.032
-Incidence density, *n* per 100 patients		8.2	2.4	0.012
*A. baumannii* BSI	32 (50.8)	17 (38.6)	15 (78.9)	0.003
-Incidence density, *n* per 100 patients		6.4	9	ns
*P. aeruginosa* BSI	5 (7.9)	5 (11.4)	0	ns
Source of BSI				ns
- Lung	31 (49.2)	21 (47.7)	10 (52.6)	
- Urine	5 (7.9)	5 (11.4)	0	
- Abdomen	2 (3.2)	2 (4.5)	0	
Primary BSI	25 (39.7)	16 (36.4)	9 (47.4)	ns
Source control	8 (12.7)	4 (9.1)	4 (21)	ns
**Clinical data**				
Septic shock at BSI onset, *n* (%)	25 (39.7)	12 (27.3)	13 (68.4)	0.003
PITT score on the BSI day, median (IQR)	3 (1–8)	3 (1–5)	8 (2–8)	0.002
**Treatment data**				
Early active therapy (<24 h), *n* (%)	31 (49.2)	21 (47.7)	10 (52.6)	ns
Time to definite therapy, median (IQR)	1 (1–2)	1 (1–2)	1 (1–2)	ns

{1} from chronic hepatitis to cirrhosis; {2} from moderate CKD (creatinine > 3 mg/dL) to dialysis or status post kidney transplant; {3} APACHE II score at ICU admission; {4} pre-BSI infections: an infection before BSI onset. No-CoV: pre- COVID-19 pandemic period; CoV: during COVID-19 pandemic; MDRO: multi-drug-resistant organism; ns: not significant; NA: not applicable; ICU: intensive care unit; BSI: bloodstream infection; early active therapy: at least one in vitro active drug within the first 24 h.

## Data Availability

Not applicable.
